# Dynamic assessment of scapholunate ligament status by real-time magnetic resonance imaging: an exploratory clinical study

**DOI:** 10.1007/s00256-023-04466-6

**Published:** 2023-10-11

**Authors:** Lena Marie Wilms, Karl Ludger Radke, Daniel Benjamin Abrar, Jens Frahm, Dirk Voit, Simon Thelen, Dirk Klee, Jan-Peter Grunz, Anja Müller-Lutz, Sven Nebelung

**Affiliations:** 1 Department of Diagnostic and Interventional Radiology, University Dusseldorf, Medical Faculty, Moorenstrasse 5, D-40225, Dusseldorf, Germany; 2 Biomedical NMR, Max Planck Institute for Multidisciplinary Sciences, D-37077 Goettingen, Germany; 3 Department of Orthopaedics and Trauma Surgery, Medical Faculty, University Dusseldorf, D-40225 Dusseldorf, Germany; 4 Department of General Pediatrics, University Dusseldorf, Medical Faculty, University Children’s Hospital, Heinrich-Heine-University Dusseldorf, Moorenstrasse 5, Düsseldorf, Germany; 5 Department of Diagnostic and Interventional Radiology, University Hospital Wurzburg, D-97080 Würzburg, Germany; 6 Department of Diagnostic and Interventional Radiology, University Hospital Aachen, D-52074 Aachen, Germany

**Keywords:** Magnetic resonance imaging, Scapholunate ligament tear, Carpal instability, Real-time, FLASH, Deep learning, Dynamic instability

## Abstract

**Objective:**

Clinical-standard MRI is the imaging modality of choice for the wrist, yet limited to static evaluation, thereby potentially missing dynamic instability patterns. We aimed to investigate the clinical benefit of (dynamic) real-time MRI, complemented by automatic analysis, in patients with complete or partial scapholunate ligament (SLL) tears.

**Material and methods:**

Both wrists of ten patients with unilateral SLL tears (six partial, four complete tears) as diagnosed by clinical-standard MRI were imaged during continuous active radioulnar motion using a 1.5-T MRI scanner in combination with a custom-made motion device. Following automatic segmentation of the wrist, the scapholunate and lunotriquetral joint widths were analyzed across the entire range of motion (ROM). Mixed-effects model analysis of variance (ANOVA) followed by Tukey’s posthoc test and two-way ANOVA were used for statistical analysis.

**Results:**

With the increasing extent of SLL tear, the scapholunate joint widths in injured wrists were significantly larger over the entire ROM compared to those of the contralateral healthy wrists (*p*<0.001). Differences between partial and complete tears were most pronounced at 5°–15° ulnar abduction (*p*<0.001). Motion patterns and trajectories were altered. Complete SLL deficiency resulted in complex alterations of the lunotriquetral joint widths.

**Conclusion:**

Real-time MRI may improve the functional diagnosis of SLL insufficiency and aid therapeutic decision-making by revealing dynamic forms of dissociative instability within the proximal carpus. Static MRI best differentiates SLL-injured wrists at 5°–15° of ulnar abduction.

**Supplementary Information:**

The online version contains supplementary material available at 10.1007/s00256-023-04466-6.

## Introduction

The scapholunate ligament (SLL) is an essential stabilizer of the proximal wrist, maintaining the biomechanical stability of the carpus during motion and loading [1]. Injuries to the SLL, caused primarily by traumatic hyperextension [2], constitute the most common cause of carpal instability [3]. Carpal instability, in turn, may lead to chronic wrist pain, malalignment, functional disability, degenerative joint disease, and irreversible joint damage [4, 5]. While static carpal instability can be readily diagnosed by signs of carpal dissociation at rest, e.g., in conventional radiographs or computer tomography studies, more complex dynamic patterns remain diagnostically challenging [3]. Such dynamic instability patterns manifest as carpal malalignment only during active wrist motion or loading [3], thus requiring a dynamic examination to become apparent. Dynamic imaging modalities such as stress radiography or wrist cineradiography suffer from numerous limitations, however, including exposure to ionizing radiation, lack of direct soft tissue visualization, and the fact that 2D projections are used to assess 3D structures. These limitations have led to only moderate diagnostic accuracy and reproducibility [6] and prevented widespread clinical adoption [2, 3, 7].

In contrast to the aforementioned modalities, magnetic resonance imaging (MRI) is the standard clinical imaging technique for soft tissue evaluation and the only non-invasive examination technique capable of directly visualizing the intrinsic stabilizers of the wrist [2]. In clinical practice, MR images are acquired in static configurations with the wrist immobilized to prevent motion artifacts. Consequently, scapholunate dissociation may be masked in patients with SLL injuries [2, 3], rendering the detection of dynamic instability patterns impossible [8]. Additionally, acute SLL tears are often characterized by extensive soft tissue edema challenging the evaluation of structural integrity [2]. However, a timely diagnosis is crucial for appropriate therapeutic decision-making because patients with acute SLL tears should undergo surgery within 6 weeks after trauma to restore physiological carpal kinematics [3] and prevent irreversible joint damage [9].

In contrast to the established clinical-standard, fast MRI techniques allow for dynamic imaging during motion [10–13]. While pioneering feasibility studies investigated active wrist motion under simultaneous MR imaging [13–15], these studies focused more on technical aspects than clinical applicability. Whether real-time MRI introduces an actual benefit for patients remains unanswered to date. To explore the technique’s clinical potential, our study aimed to visualize, parameterize, and quantify motion patterns and trajectories alongside configurational changes of the proximal carpus in patients with SLL tears. To this end, an established imaging setup to standardize real-time MR image acquisition and post-processing [16] was used to assess configurational changes of the carpus in a patient cohort.

We hypothesized that real-time MRI is sufficiently sensitive to detect differences in scapholunate and lunotriquetral joint widths (i) intra-individually between the injured and contralateral (healthy) wrists and (ii) inter-individually between wrists with partial and complete SL tears as dynamic markers of configurational wrist changes and carpal (in)stability.

## Material and methods

### Study population and design

The present study was designed as a prospective clinical imaging study on patients with SLL tears diagnosed earlier on direct MR arthrography. Before the study, institutional review board approval (Ethical Committee, Medical Faculty, Heinrich-Heine-University, Düsseldorf, Germany, study number 2019-590) and individual written informed consent were obtained. The minimum patient number was determined as 10 based on the initial four patients, using dedicated online software (http://www.statstodo.com) and the following statistical parameters: power 0.8; probability of type-I-error 0.01; assumed effect size 1.4 [defined as the mean paired difference divided by the expected standard deviation], two-tailed procedure.

Consequently, ten patients (male: 8; female: 2; mean age: 41.2 ± 18.1 years, age range: 24 – 66 years) with partial or complete SLL tears were included (Table 1). Isolated tears of the dorsal or volar component of the SLL complex, defined as a discontinuous appearance of the ligament and consecutive entry of contrast agent, were defined as partial SLL tears. High signal intensity within individual ligament components was not considered diagnostic of a partial SLL tear. Partial SLL tears (*n* = 6 [3 right, 3 left]) involved the dorsal and volar components in 4 and 2 patients. In contrast, complete SLL tears (*n* = 4 [2 right, 2 left) were presumed when both components appeared discontinuous.

**Table 1:** Patient demographics and clinical MR imaging information

Patient	Sex	Age [years]	Side of injured wrist	Scapholunate ligament tear	Type of treatment	Time from injury to real-time MRI [months]	Time from MR arthrography to real-time MRI [weeks]
1	male	46	left	partial-dorsal	conservative	32	4
2	male	66	right	complete	conservative	35	26
3	male	42	right	partial-volar	conservative	21	4
4	male	52	right	complete	surgical	17	2
5	male	56	left	partial-dorsal	conservative	18	11
6	female	27	left	partial-dorsal	conservative	34	33
7	male	47	right	partial-dorsal	conservative	7	7
8	male	43	left	complete	surgical	14	2
9	female	24	right	partial-volar	conservative	11	3
10	male	50	left	complete	conservative	16	5

SL Ligament Tear: “partial-dorsal” and “partial-volar” refer to the isolated tear of the dorsal or volar component of the SL ligament. Abbreviation: MRI – Magnetic Resonance Imaging

The real-time MRI studies were performed 21 ± 10 months (range 7–35 months) after the initial injury and 10 ± 11 weeks (range 2–33 weeks) after the direct MR arthrography. At the time of the real-time MRI studies, all patients still suffered from mild or moderate chronic wrist pain. To ensure the absence of tear progression or re-injury to the symptomatic wrist, all patients underwent a thorough medical review of their clinical history, a physical examination to detect any potential indications of recurrent injury to the affected wrist, and a thorough imaging evaluation utilizing morphologic, static MR imaging.

Each patient’s contralateral wrist served as the reference. Consequently, acute or chronic wrist pain or a history of wrist trauma or surgery were defined as exclusion criteria. In the following, “injured_partial_” and “injured_complete_” refer to the wrists of patients with partial and complete SLL tears, respectively, while “control_partial_” and “control_complete_” refer to the respective (healthy) contralateral wrists.

### MR imaging

**Fig. 1:**
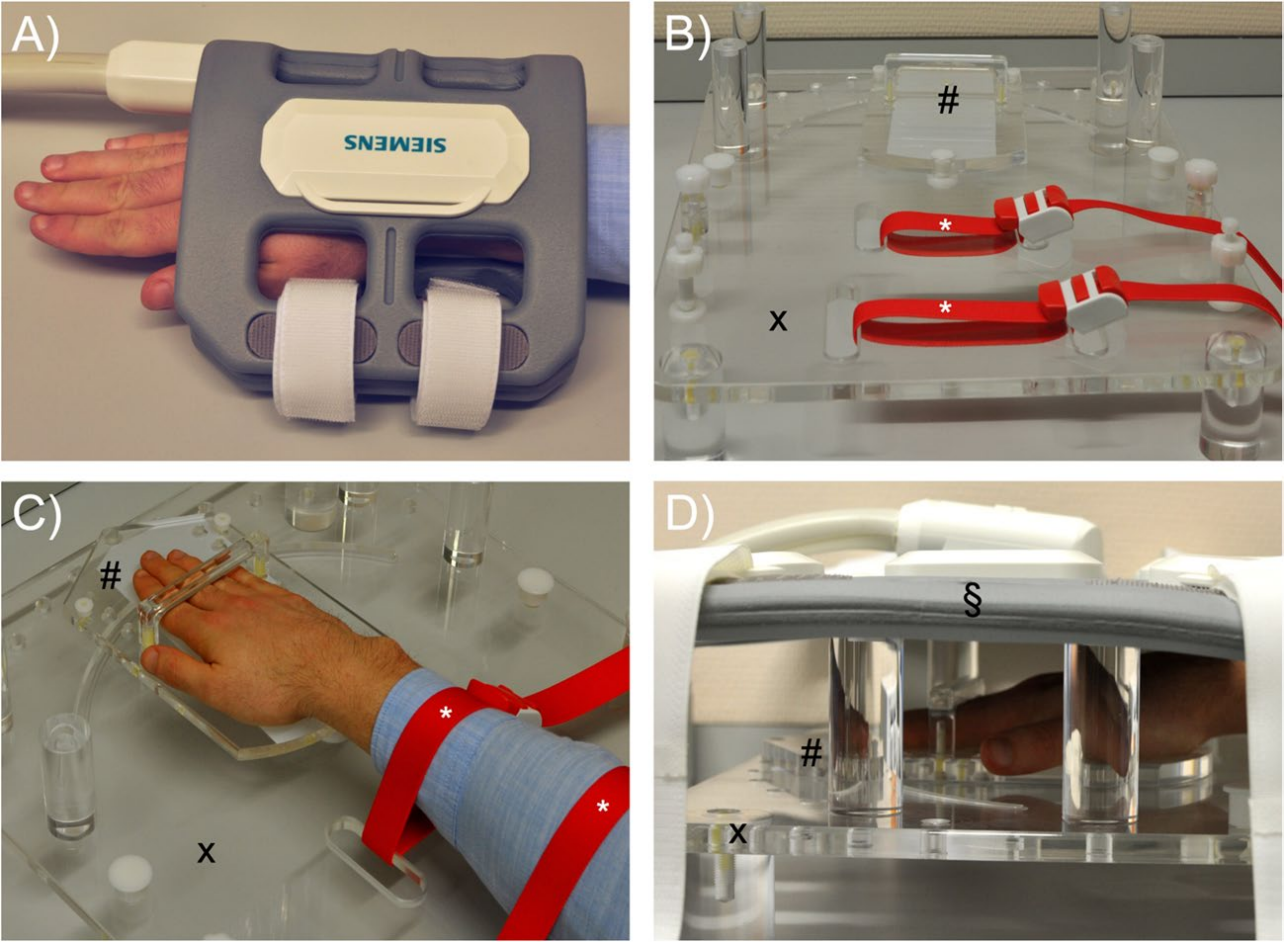
Details of the MR imaging setups. **A**) During static imaging, morphologic sequences were acquired with a standard 4-channel flex coil centered around the wrist. **B-D**) Setup for real-time MRI measurements: The MRI-compatible motion device is shown without (**B**) and with (**C**) a patient’s right forearm and hand. The hand was positioned on the mobile sliding plate (#) and, thus, guided along a pre-defined semi-circular range on the immobile base plate (x). Tourniquets were used to immobilize the forearm (*). The loaded and operational device (**D**) was covered with an 18-channel body coil (§) placed on top of the transparent spacers and centered around the wrist. A 32-channel spine matrix coil was positioned underneath the device – left out for clarity

In the same session, all patients underwent morphologic standard (i.e., static) and real-time (i.e., dynamic) MRI of both wrists, i.e., the injured and the healthy side. MR images were acquired on a clinical 1.5T scanner (MAGNETOM Avanto^fit^, Siemens Healthineers, Erlangen, Germany) using two different imaging setups (Fig. 1).

First, static imaging was performed using a 4-channel flex coil (Flex Coil small, Siemens Healthineers) wrapped around the wrist for an optimized signal-to-noise ratio (Fig. 1A). Patients were placed in a prone, head-first position with the examined arm stretched out (“superman position”). Coronal, axial, and sagittal proton density-weighted (PDw) fat-saturated (fs) sequences and axial T2-weighted sequences were obtained for morphologic evaluation (Table 2).

**Table 2:** Acquisition parameters of MRI sequences

	PDw fs	PDw fs	PDw fs	T2w	Radial real-time
Sequence Type	2D TSE	2D TSE	2D TSE	2D TSE	FLASH
Orientation	cor	sag	ax	ax	cor
Repetition time [ms]	3200	3200	3200	5820	4.5
Echo time [ms]	33	33	33	114	2.5
Turbo spin-echo factor	10	10	10	10	n/a
Field of view [mm]	120 x 120	120 x 120	120 x 120	150 x 150	168 x 168
Image matrix [pixels]	256 x 256	256 x 256	256 x 256	384 x 384	168 x 168
Flip angle [°]	180	180	180	15	7
Slices [n]	20	20	20	20	1
Pixel size [mm/pixel]	0.5 x 0.5	0.5 x 0.5	0.5 x 0.5	0.4 x 0.4	1.0 x 1.0 (*)
Slice thickness / Gap [mm]	2 / 0.2	2 / 0.2	2 / 0.2	3 / 0.6	6 / n/a
Duration [min:sec]	2:54	2:54	2:54	3:10	0:30 (**)

PDw – Proton Density-weighted; TSE – turbo spin echo; fs – fat-saturated; cor – coronal; ax – axial; sag – sagittal; FLASH – fast-low-angle shot; GRE – gradient echo; n/a – not applicable. (*) The acquired pixel size was 1.0 × 1.0 mm, while the reconstructed pixel size following interpolation was 0.5 × 0.5 mm. (**) Two full cycles were acquired

Second, for dynamic imaging by real-time MRI, wrists were positioned on a custom-made MRI-compatible motion device that guides the wrist along a pre-defined semi-circular range and standardizes the motion plane during active radioulnar motion (Fig. 1B) [16]. The hand was placed on a mobile sliding plate with an under-surface covered with synthetic polytetrafluoroethylene (“Teflon”, DuPont, Wilmington, DE, US) to reduce friction. The wrist was centered on the pivot point, allowing for a semi-circular motion of 60° on each side. Two tourniquets were used to attach the forearm to the device, thus decreasing adaptive forearm motion (Fig. 1C). The loaded motion device was positioned centrally in the scanner’s bore with an 18-channel body coil (Body 18, Siemens Healthineers) centered on the radiocarpal joint and placed on top of the device (Fig. 1D). A 32-channel spine matrix coil (Direct connect spine 32, Siemens Healthineers) was positioned underneath the device.

Other than the SLL tears described above, no additional ligamentous and osseous injuries of the carpus were found in patients. D.B.A. (board-certified clinical radiologist with six years of experience in musculoskeletal imaging) assessed all static MRI examinations performed in this study while remaining blinded to the diagnosis obtained from the previously acquired direct MR arthrography, the surgical findings, and also to the information which wrist served as the healthy control and which wrist was the injured wrist during the interpretation process, thereby ensuring an unbiased assessment of the static MR images.

### Real-time MRI

For real-time MR imaging during active radioulnar abduction, 300 images and 15 pre-scans were acquired of each patient’s wrists in 30 s. We used a temporally optimized radial FLASH real-time MRI sequence validated before [16] that is characterized by pronounced radial undersampling, resulting in a temporal resolution of 95 ms per image (Table 2). Patients were instructed to avoid out-of-plane motion, including pro- or supination. All patients were instructed to continuously perform active radioulnar motion at a standard frequency of 15 s per complete cycle, i.e., from maximum radial abduction to maximum ulnar abduction and vice versa. Two complete cycles were acquired per patient. The coronal image plane was defined in the neutral position and aligned with the center of the scapholunate joint as defined on sagittal and axial scout views. On average, magnet time was 30 minutes per patient, i.e., 15 min per wrist.

### Quantitative analysis of the scapholunate and lunotriquetral joints

For the evaluation of the dynamic MR images, we employed a previously developed and validated framework to quantify scapholunate and lunotriquetral joint widths [16]. This framework employs an automated process for the semantic segmentation of the wrist and forearm, utilizing a convolutional neural network. Based on the segmentation outlines, advanced post-processing techniques are applied to determine the joint widths as a function of wrist angle. Notably, owing to the entirely automated nature of this process, no radiologist was engaged in the quantitative analysis of the dynamic MR images, thus ensuring an unbiased assessment of the dynamic MR images.

First, using a Gaussian anti-aliasing filter, the images were bi-quadratically interpolated from 168×168 pixels to 336×336 pixels. Then, the outlines of the distal radius, distal ulna, scaphoid, lunate, triquetrum, hamate, capitate, trapezium, and trapezoid were automatically segmented using a U-net based convolutional neural network. The segmented outlines of the scaphoid, lunate, and triquetrum were smoothed and connected using a centreline to determine scapholunate and lunotriquetral joint widths automatically. The scapholunate joint width was determined as the distance between the intersections of the centreline with the scaphoid’s ulnar cortex and the lunate’s radial cortex. Correspondingly, the lunotriquetral joint width was determined as the distance between the intersections of the centreline with the lunate’s ulnar cortex and the triquetrum’s radial cortex.

Additionally, the wrist angle was determined using minimal bounding boxes around the distal carpal bones, i.e., hamate, capitate, trapezium, and trapezoid, and around the bone contours of the distal radius and the distal ulna. Subsequently, the angle between the centers of the two bounding boxes was quantified as the wrist angle [16]. The wrist angles were grouped into 5° intervals for visualization and comprehensibility.

### Statistical analysis

Statistical analyses were performed by L.M.W. using GraphPad Prism (v9.5.0, San Diego, CA, US). Data are presented as mean ± standard deviations. Differences (Δ) in joint widths were determined between the injured wrist and the same patient’s contralateral healthy wrist as a function of wrist position (intraindividual). Absolute joint widths were comparatively evaluated between patients (interindividual). Assuming normal distributions of the scapholunate and lunotriquetral joint widths, the respective widths were compared group-wise across the entire range of motion (ROM) using parametric tests. Mixed effects analysis-of-variance (ANOVA) was used for the group-wise analysis of joint widths across the entire ROM. In contrast, two-way ANOVA was used for the subsequent group-wise comparison of injured and healthy wrists (intraindividual). Mixed-effects ANOVA was also used for comparing joint widths between groups at distinct ROM intervals (interindividual), followed by select Tukey’s multiple comparison posthoc tests. To reduce the number of statistically significant yet (most likely) clinically irrelevant findings, we set the significance level to *p*≤0.01. Multiplicity-adjusted *p*-values are reported to account for the multiple comparisons involved.

## Results

**Fig. 2:**
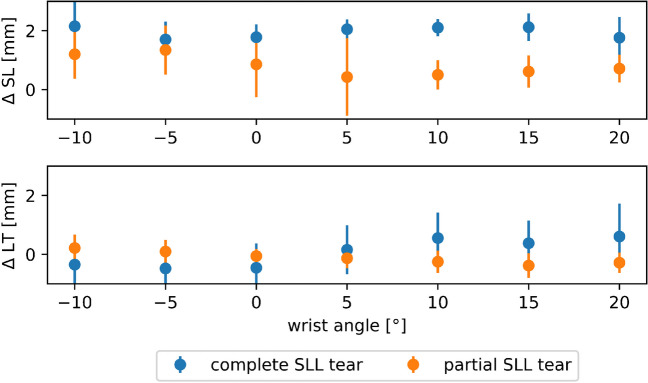
Intraindividual differences in scapholunate and lunotriquetral joint widths as a function of wrist position and scapholunate ligament condition. For the scapholunate (SL) and lunotriquetral (LT) joint, absolute differences in joint widths (Δ) were determined in each wrist position between the injured wrist and the same patient’s healthy contralateral wrist (intraindividual). Partial scapholunate ligament (SLL) tears (orange circles) and complete SLL tears (blue circles) were compared. SL and LT joint widths are plotted over the entire range of motion, i.e., from maximum radial abduction (-10°) to maximum ulnar abduction (20°). Data are means ± standard deviation [mm]

**Fig. 3:**
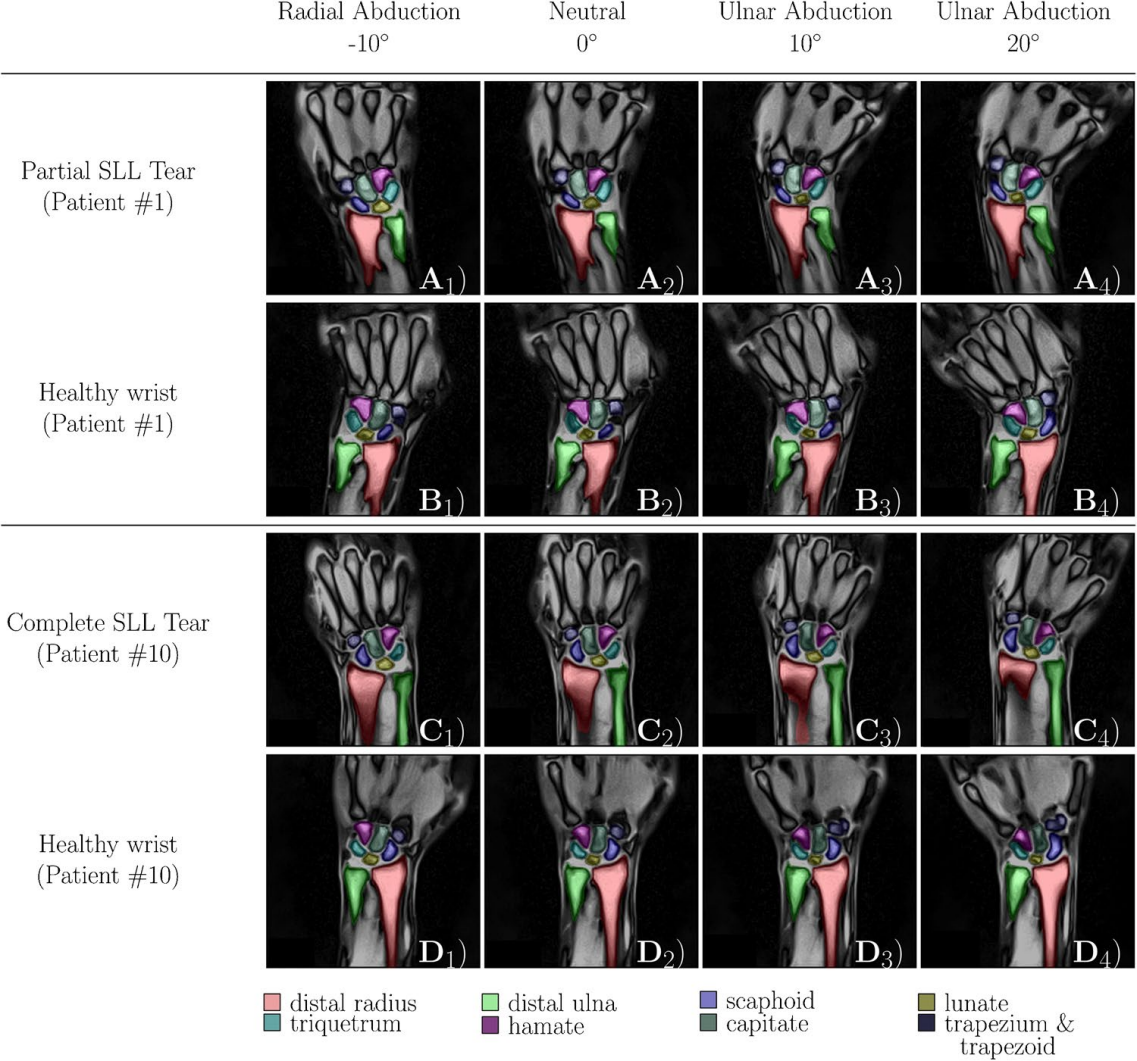
Visualization of injured and healthy wrists in different wrist positions as a function of scapholunate ligament condition. Displayed are selected real-time MR images in two exemplary patients. Patient #1 had a tear of the dorsal scapholunate ligament (SLL). His injured (A_1_-A_4_) and contralateral healthy wrists (B_1_-B_4_) are shown. Patient #10 had a complete SLL tear (C_1_-C_4_, D_1_-D_4_). The forearm and carpal bones were segmented automatically and color-coded as detailed below

The mean scapholunate and lunotriquetral joint widths as a function of wrist position and SLL condition versus the respective intra-individual control wrist are detailed in Fig. 2. The mean absolute joint widths as a function of wrist position and SLL condition are indicated in Tables 3 and 4. The corresponding posthoc test results are given in Supplementary Tables 1 and 2. Figure 3 visualizes these changes as a function of wrist position and SLL condition. Scapholunate joint widths were broader in SLL-injured wrists than in control wrists. In both patients who underwent surgical intervention, the presence of complete SLL tears could be confirmed.

**Table 3:** Absolute scapholunate joint width as a function of wrist position and scapholunate ligament condition. For each patient and control, absolute values of scapholunate joint widths [mm] were determined as a function of scapholunate ligament condition and wrist position across the entire range of motion (ROM), i.e., from maximum radial abduction (-10°) to maximum ulnar abduction (+20°). “Injured” refers to the injured wrist, while “control” refers to the contralateral healthy wrist. Within each group, the scapholunate joint widths were compared over the entire ROM by means of mixed effects analysis-of-variance (ANOVA) with respective *p*-values displayed in a row-wise manner (‡). Among injured and healthy wrists, scapholunate joint widths were compared over the entire ROM using two-way ANOVA, and the respective *p*-values are organized row-wise, too (#). For inter-individual comparisons at distinct ROM intervals, scapholunate joint widths were compared using mixed-effects ANOVA followed by Tukey’s multiple comparison posthoc tests. The respective *p*-values are organized column-wise (†). Mean ± standard deviation. Significant findings are indicated in bold type. Sequential numbers in square brackets indicate the corresponding posthoc details as given in Supplementary Table 1. Abbreviations: ROM – range-of-motion; SL – scapholunate; ANOVA – analysis-of-variance

	SL Joint Width[mm]								*p*-values	
	Entire	-10	-5	0	+5	+10	+15	+20	Overall(‡)	Control vs. Injured (#)
Control_partial_	1.77±0.93	1.42±0.87	1.56±0.87	1.79±1.01	2.16±1.02	2.00±0.86	1.70±0.82	1.75±0.90	<0.001	<0.001
Injured_partial_	3.12±1.28	3.01±1.12	3.18±1.30	3.34±1.12	3.10±1.23	3.03±1.50	2.36±1.23	3.13±1.59	<0.001	
Control_complete_	1.67±0.92	3.42±0.84	2.37±0.90	1.61±0.96	1.09±0.56	1.28±0.57	1.32±0.68	1.32±0.68	<0.001	<0.001
Injured_complete_	3.39±1.14	3.43±1.28	2.87±1.55	3.33±1.82	3.84±1.53.	3.91±1.32	3.58±1.07	3.58±1.07	<0.001	
*p*-values†	<0.001 [1]	<0.001 [2]	<0.001 [3]	<0.001 [4]	<0.001 [5]	<0.001 [6]	<0.001 [7]	<0.001 [8]

**Table 4:** Absolute lunotriquetral joint width as a function of wrist position and scapholunate ligament condition. For each patient and control, absolute values of lunotriquetral joint widths [mm] were determined as a function of scapholunate ligament condition and wrist position across the entire range of motion, i.e., from maximum radial abduction (-10°) to maximum ulnar abduction (+20°). Table organization as in Table 3. Sequential numbers in square brackets indicate the corresponding posthoc details as given in Supplementary Table 2. Abbreviations: LT – lunotriquetral

	LT Joint Width[mm]								*p*-values	
	Entire	-10	-5	0	+5	+10	+15	+20	Overall‡	Control vs. Injured (#)
Control_partial_	1.33±0.66	2.00±0.50	1.14±0.69	1.24±0.50	1.34±0.67	1.46±0.69	1.44±0.67	1.54±0.70	<0.001	0.891
Injured_partial_	1.30±0.66	1.34±0.69	1.42±0.68	1.28±0.68	1.42±0.82	1.15±0.45	1.13±0.54	1.19±0.48	<0.001	
Control_complete_	1.40±0.98	1.73±0.76	2.23±1.75	1.44±1.15	1.26±0.93	1.18±0.43	1.14±0.37	1.15±0.34	<0.001	0.013
Injured_complete_	1.23±0.89	1.23±0.39	1.04±0.34	0.97±0.38	1.27±0.55	1.32±0.55	1.22±1.01	1.67±1.69	<0.001	
*p*-values†	<0.001 [9]	<0.001 [10]	<0.001 [11]	<0.001 [12]	0.269	<0.001 [13]	<0.001 [14]	<0.001 [15]

### Changes in the scapholunate joint

In healthy controls, the scapholunate joint width remained stable at around 2 mm over the entire ROM. In contrast, in partially and completely SLL-injured patients, the scapholunate joint widths were larger and variably increased with wrist position. Consequently, intra-individual differences in scapholunate joint widths (versus the corresponding healthy wrists) were significant in completely and partially SL-injured patients throughout the entire ROM. In partially SLL-injured patients, the scapholunate joint widths were significantly larger than in the contralateral healthy wrists (injured_partial_ = 3.12±1.28 mm vs. control_partial_ = 1.77±0.93 mm; *p*<0.001). Off note, their scapholunate joint widths were largest in the neutral position. In completely SLL-injured patients, the SL joint widths were even larger over the entire ROM (injured_complete_ = 3.39±1.41 mm vs. control_complete_ = 1.67±0.92 mm; *p*<0.001) with a maximum at 5° to 10° ulnar abduction.

In both partially and completely SLL-injured patients, the scapholunate joint widths significantly differed over the entire ROM (*p*<0.001). Inter-individual comparisons between partially and completely SLL-injured patients indicated significant differences for 5 to 15° ulnar abduction (*p*<0.001), while no significant differences were observed for the neutral position or radial abduction (Supplementary Table 1).

### Changes in the lunotriquetral joint

Intra-individual comparison revealed no significant differences in the lunotriquetral joint widths both in partially (injured_partial_ = 1.30±0.66 mm vs. control_partial_ = 1.33±0.66 mm [*p*=0.891]) or completely SLL-injured patients (injured_complete_ = 1.23±0.89 mm vs. control_complete_ = 1.40±0.98 mm; [*p*=0.013]).

While in partially SLL-injured patients, the lunotriquetral joint widths remained relatively stable throughout the entire ROM, complex changes in lunotriquetral joint widths were found in the presence of complete SLL tears. Lunotriquetral joint widths increased during ulnar abduction and decreased during radial abduction. Significant differences in lunotriquetral joint widths between partially and completely SLL-injured patients were found for the neutral position and at 5° radial abduction (*p*<0.001) (Supplementary Table 2).

## Discussion

The most important findings of this study are that (i) the configuration of the proximal carpus is dynamically altered across the entire ROM, (ii) the extent of a SLL tear is best differentiated in slight ulnar abduction, and (iii) scapholunate and lunotriquetral joint widths need to be considered in conjunction to fully understand the complex relationship between structural and functional integrity of the carpus.

In healthy control wrists, scapholunate joint widths were consistently measured as 2 mm or lower, which aligns well with the literature [3, 17]. In contrast, scapholunate joint widths of SLL-injured patients were not homogeneous across the entire ROM but associated with the wrist angle. Whereas scapholunate SL joint widths decreased with increasing ulnar abduction in partially SLL-injured wrists, scapholunate joint widths were determined to be largest between 5° and 10° ulnar abduction in complete SLL tears. Importantly, in vivo evidence suggests that scaphoid and lunate motion occurs along three principal axes, i.e., flexion-extension, supination-pronation, and radioulnar abduction, while being greatest from the neutral position to 20° ulnar abduction and smallest in radial abduction [18]. During motion along the coronal plane, i.e., radial and ulnar abduction, the scaphoid is also subject to rotation and translation [19, 20]. Consequently, the SLL stabilizes the scaphoid’s attachment to the lunate. If entirely insufficient, the motion amplitude increases, and scapholunate dissociation occurs, particularly between 5° and 10° of ulnar abduction. Additional factors that may affect the configuration of the scapholunate joint are the overarching carpal alignment and configuration, laxity, and the patient’s handedness.

Our results suggest, that ulnar abduction between 5 and 15° allows for the most accurate differentiation of healthy wrists, partial, and complete SLL tears. Consequently, if a clinical wrist MRI scan is conducted statically, the wrist should be examined at 5 to 15° of ulnar abduction to assess SLL integrity, provided the wrist coil allows this position.

Compared to the healthy wrists, the scapholunate joint widths were significantly wider in partially and completely SLL-injured wrists, which is consistent with the literature: Increased scapholunate joint widths ≥ 3 mm are indicative of scapholunate instability [21, 22]. Our data also confirmed the intuitive notion that scapholunate joint widths are associated with the extent of the SLL tear. We found moderate increases in partial SLL tears and larger increases in complete SLL tears: The complete SLL tear inevitably led to excessive rotational, translational, and deviational instability of the scaphoid. Additionally, it caused the scapholunate joint to be substantially wider in ulnar abduction (where the scaphoid undergoes extension) than in radial abduction (where the scaphoid undergoes flexion). A large body of scientific evidence supports these observations [9, 19, 20], in particular, in situ data based on serial transections of human cadaveric specimens [19] and in vivo data based on two patients with suspected SLL tears [23]. Consequently, there is a clear connection between the scapholunate joint width pattern under motion and the extent of SLL damage.

While lunotriquetral joint widths remained stable in partially SLL-injured wrists across the entire ROM, completely SLL tears entailed substantially larger lunotriquetral joint widths in ulnar abduction and smaller lunotriquetral joint widths in radial abduction. Variable lunate trajectories may explain this finding. In complete scapholunate dissociation, the lunate undergoes rotational and deviational motion [24]. As outlined above, insufficiency of the SLL decreases radial traction on the lunate, which is pulled towards the triquetrum by the intact lunotriquetral ligament and follows the natural motion of the triquetrum into extension [2]. In situ data indicate larger lunate extension in ulnar abduction than in radial abduction in completely SLL-injured wrists [25]. Correspondingly, the smaller lunotriquetral joint widths in radial abduction may be secondary to aberrant fixation and motion restriction.

Direct MR arthrography, has proven effective in assessing intracarpal pathologies by enhancing the visibility of intra-articular structures such as ligaments and cartilage, allowing for the identification of subtle tears and small lesions [26]. However, there is a potential drawback to this technique, as it may detect ligament defects with minimal clinical or biomechanical significance, potentially leading to unnecessary treatment [26]. In contrast, real-time MRI offers a non-invasive and dynamic imaging approach during active wrist motion, offering a comprehensive evaluation of joint function. Its dynamic assessment capabilities make it a valuable adjunct to the diagnostic armamentarium, complementing the standard static MRI exam for evaluating wrist instability and ligamentous tears. By visualizing joint structures in real-time during active motion, real-time MRI enhances sensitivity for detecting dynamic wrist instability as a surrogate of clinically and biomechanically relevant ligament tears and dynamic abnormalities that static imaging might miss. This, in turn, might lead to more targeted treatment strategies. Therefore, real-time MRI’s dynamic assessment capabilities offer a unique perspective on joint function, thus providing critical knowledge on wrist stability that could improve diagnostic accuracy and patient care.

Due to its exploratory nature, the present study cannot establish the diagnostic value of real-time MRI and time-resolved motion analyses in SLL tears. Yet, it is conceivable that this approach may serve as a diagnostic adjunct in otherwise inconclusive patients, where static MRI approaches fail to yield a definitive diagnosis.

Some limitations need to be recognized. First, patient numbers were limited, and larger studies need to confirm our findings. Second, the time lag between the initial diagnosis (following injury) and the real-time MRI study was substantial, so variable ligament scarring and healing processes were present but not accounted for in this exploratory clinical study. Yet, to enhance accuracy in diagnosing SLL tears, it is advisable to perform imaging within 6 weeks following injury. To address this limitation, future clinical studies should prioritize conducting more immediate evaluations of patients to gain valuable insights into the dynamic processes of acute SLL tears, ultimately enhancing the accuracy and applicability of diagnostic approaches in such cases. Third, the mean time interval between the direct MR arthrography and dynamic wrist MRI was approximately 10 weeks, with one case extending up to 33 weeks. Through a thorough medical examination and morphologic, static MRI, we attempted to rule out potential tear progression. Further research with shorter time intervals would be beneficial in gaining a more comprehensive understanding of the dynamics of SLL tears. Fourth, it is important to acknowledge that surgical confirmation of SLL tears was only performed in two patients included in this study. While the use of real-time MRI provided valuable insights into the dynamic assessment of SLL tears, the absence of surgical correlation limits the ability to definitively establish the accuracy of the MRI findings. Having surgical confirmation in all patients would have allowed for a direct comparison between the real-time MRI results and the intraoperative findings, thereby enhancing the validity and reliability of this diagnostic method. Future studies should therefore consider incorporating surgical confirmation in a larger cohort to validate the effectiveness and clinical utility of real-time MRI in the assessment of SLL tears. This step would strengthen the evidence and enhance the clinical application of real-time MRI as a valuable tool in the diagnosis and management of wrist pathologies. Fifth, the real-time MRI sequence was only acquired as a single cross-sectional image series (movie). Thereby, complex 3D geometric alterations of the carpus following rotational, translational, and deviational instability may have been missed [9]. Consequently, future studies must define a sensible balance between acquisition time (e.g., following multi-slice approaches) and sufficiently high temporal and spatial resolution. Additional aspects worth considering are patient comfort, measurement accuracy and reproducibility, as well as the model’s implementation in the clinic. In wrist-injured patients, discomfort, pain, swelling, and other bone and soft tissue pathologies may alter and reduce wrist motion. Fifth, we did not further differentiate the partial SLL tears based on the involvement of the ligament’s volar or dorsal component. Both components are morphologically and functionally different. With its thickness of 3 mm, the dorsal component is the strongest and controls flexion and extension, while the volar component is only 1 mm thick and controls rotation [27]. Consequently, larger patient numbers with partial SLL tears must be studied to overcome our cohort’s heterogeneity.

In conclusion, this exploratory study demonstrates the potential of real-time MRI in diagnosing scapholunate instability by examining dynamic alterations in the proximal carpus. Scapholunate and lunotriquetral joint widths may be surrogate markers of functional carpal instability. When using static MRI in the clinical routine, the optimal wrist position to differentiate partially and completely SL-injured wrists is 5° to 15° of ulnar abduction. Once validated in larger studies, diagnostic accuracy could be improved and provide the basis for enhanced therapeutic decision-making and monitoring of treatment outcomes.

### Supplementary information

ESM 1 (DOCX 168 kb)

## Data Availability

Data can be provided by the authors upon reasonable request.

## References

[1] Rajan PV, Day CS (2015). Scapholunate Interosseous Ligament Anatomy and Biomechanics. J Hand Surg..

[2] Schmitt R, Fröhner S, Fodor S, Christopoulos G, Kalb KH (2006). Early radiological diagnostics for scapholunate dissociation (SLD). Radiologe..

[3] Andersson JK (2017). Treatment of scapholunate ligament injury: Current concepts. EFORT Open Rev..

[4] Shih J-T, Hou Y-T, Lee H-M, Tan C-M (2000). Chronic triangular fibrocartilage complex tears with distal radioulna joint instability: A new method of triangular fibrocartilage complex reconstruction. J Orthop Surg (Hong Kong)..

[5] Watson HK, Weinzweig J, Zeppieri J (1997). The natural progression of scaphoid instability. Hand Clin..

[6] Sulkers GSI, Schep NWL, Maas M, van der Horst CM, Goslings JC, Strackee SD (2014). The diagnostic accuracy of wrist cineradiography in diagnosing scapholunate dissociation. J Hand Surg Eur..

[7] Andersson JK, Andernord D, Karlsson J, Fridén J (2015). Efficacy of Magnetic Resonance Imaging and Clinical Tests in Diagnostics of Wrist Ligament Injuries: A Systematic Review. Arthroscopy..

[8] Kitay A, Wolfe SW (2012). Scapholunate instability: current concepts in diagnosis and management. J Hand Surg Am..

[9] Konopka G, Chim H (2018). Optimal management of scapholunate ligament injuries. Orthop Res Rev..

[10] Frahm J, Haase A, Matthaei D (1986). Rapid NMR imaging of dynamic processes using the FLASII technique. Magn Resonance in Med..

[11] Uecker M, Zhang S, Voit D, Karaus A, Merboldt K-D, Frahm J (2010). Real-time MRI at a resolution of 20 ms. NMR Biomed..

[12] Tsao J, Boesiger P, Pruessmann KP (2003). k-t BLAST and k-t SENSE: dynamic MRI with high frame rate exploiting spatiotemporal correlations. Magn Reson Med..

[13] Shaw CB, Foster BH, Borgese M, Boutin RD, Bateni C, Boonsri P, u. a. (2019). Real-time three-dimensional MRI for the assessment of dynamic carpal instability. PLoS ONE..

[14] Boutin RD, Buonocore MH, Immerman I, Ashwell Z, Sonico GJ, Szabo RM (2013). Real-Time Magnetic Resonance Imaging (MRI) during Active Wrist Motion—Initial Observations. Hug F, Herausgeber. PLoS ONE..

[15] Henrichon SS, Foster BH, Shaw C, Bayne CO, Szabo RM, Chaudhari AJ, u. a. (2020). Dynamic MRI of the wrist in less than 20 seconds: normal midcarpal motion and reader reliability. Skeletal Radiol..

[16] Radke KL, Wollschläger LM, Nebelung S, Abrar DB, Schleich C, Boschheidgen M, u. a. (2021). Deep Learning-Based Post-Processing of Real-Time MRI to Assess and Quantify Dynamic Wrist Movement in Health and Disease. Diagnostics (Basel)..

[17] Kindynis P, Resnick D, Kang HS, Haller J, Sartoris DJ (1990). Demonstration of the scapholunate space with radiography. Radiology..

[18] Tang JB, Xu J, Xie RG (2011). Scaphoid and lunate movement in different ranges of carpal radioulnar deviation. J Hand Surg Am..

[19] Short WH, Werner FW, Green JK, Sutton LG, Brutus JP (2007). Biomechanical evaluation of the ligamentous stabilizers of the scaphoid and lunate: part III. J Hand Surg Am..

[20] Moojen TM, Snel JG, Ritt MJPF, Venema HW, Kauer JMG, Bos KE (2002). Scaphoid kinematics in vivo. J Hand Surg Am..

[21] Chennagiri RJR, Lindau TR (2013). Assessment of scapholunate instability and review of evidence for management in the absence of arthritis. J Hand Surg Eur..

[22] Manuel J, Moran SL (2007). The Diagnosis and Treatment of Scapholunate Instability. Ortho Clin North Am..

[23] Kakar S, Breighner RE, Leng S, McCollough CH, Moran SL, Berger RA, u. a. (2016). The Role of Dynamic (4D) CT in the Detection of Scapholunate Ligament Injury. J Wrist Surg..

[24] Flores DV, Umpire DF, Mejía Gómez C, Saad T, Cerezal L, Pathria MN (2021). Carpal Instability: Anatomy, Kinematics, Imaging, and Classification. Radio Graphics..

[25] Short WH, Werner FW, Green JK, Masaoka S (2002). Biomechanical evaluation of ligamentous stabilizers of the scaphoid and lunate. J Hand Surg Am..

[26] Lee RKL, Ng AWH, Tong CSL, Griffith JF, Tse WL, Wong C, u. a. (2013). Intrinsic ligament and triangular fibrocartilage complex tears of the wrist: comparison of MDCT arthrography, conventional 3-T MRI, and MR arthrography. Skeletal Radiol..

[27] Berger RA (1996). The gross and histologic anatomy of the scapholunate interosseous ligament. J Hand Surg Am..

