# Supplementary material for: Dynamic assessment of scapholunate ligament status by real-time magnetic resonance imaging: an exploratory clinical study
Source: Skeletal Radiol. 2023 Oct 11;53(4):791–800. doi: 10.1007/s00256-023-04466-6 (PMC10858828; doi:10.1007/s00256-023-04466-6)

**Supplementary Material**

**Supplementary Table 1: Post-hoc details of group-wise comparisons of scapholunate joint widths.**

Given are the details of Tukey’s multiple comparisons tests following repeated measures ANOVA as detailed in **Table 3**. The level of significance (*p* ≤ 0.01) was further stratified as 0.01 ≥ p > 0.001 (**) and p ≤ 0.001 (***), with significant findings indicated in **bold type**.


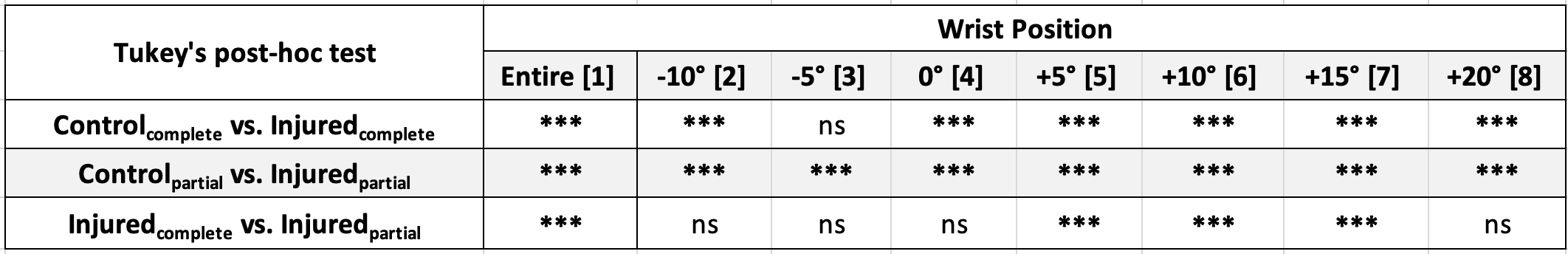


**Supplementary Table 2: Post-hoc details of group-wise comparisons of lunotriquetral joint widths.**

Given are the details of Tukey’s multiple comparisons tests following repeated measures ANOVA as detailed in **Table 4**. Table organization as in **Supplementary Table 1** above.


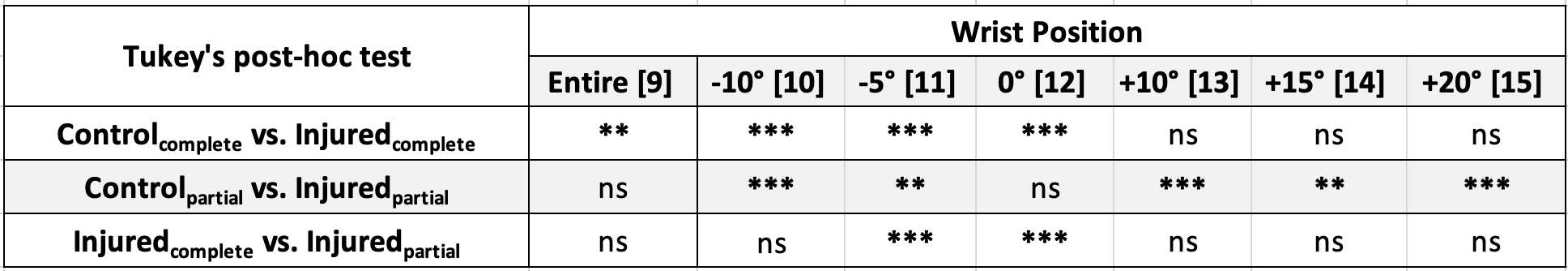

Supplement: Supplementary file 1 — (DOCX 168 kb) [file 256_2023_4466_MOESM1_ESM.docx]
